# Essay on the Fucus Helminthocorton, or Corsican Moss, in Cancer

**Published:** 1822-03-01

**Authors:** 


*An Essay on the Effects of the Fucus Helminthocorton upon Cancer, more especially in the Stage denominated Occult, &c. &c. &c.* By William Farr, Member of the Royal College of Surgeons, &c. One vol. 8vo, pp. 112. London, 1822. With a Plate.

Prona via est et eget moderamine certo.

Our readers need not imagine that we have one particle more faith in the power of "Corsican moss" over cancer, than in that of "Iceland moss" over consumption. We notice the work before us merely for the sake of alluding to some of the pretended specifics for this last formidable disease, which have, in their turn, excited the curiosity of credulous practitioners, abused the ear of the public, and, as may readily be imagined, disappointed the hopes of the suffering patient. Human life, though despised, in theory, by philosophers and enthusiasts, is yet so highly prized, in fact, by all ranks and ages, that when assailed by incurable diseases, the patient will fly from the candid physician to the lying quack, or ignorant pretender; and to ensure a trial of any nostrum, however absurd, it is merely necessary to roundly assert, without a particle of evidence except the ipse dixit of the assertor, that it is infallible. If a new and a hard name can be found for the remedy—or a juggling mysterious air given to the measure, the work is half done. Those veracious records of the times, the newspapers, proclaim the important discovery, and the system of delusion is forthwith in full operation.

Now did the nostrum-monger and "peculiar practice" man extend their fallacious promises only to those whose maladies resisted the regular practitioner, it would be exceedingly pardonable, for what can be a more terrible situation than that of corporeal suffering without prospect of relief, except by a lingering death! But the misfortune is, that thousands of people are persuaded through their own fears, and the representations of the unprincipled, that they are labouring under formidable diseases, when their complaints are really harmless, and thus they become the dupes, and too often the victims of mercenary Medicasters or barefaced Charlatans. We have reason to believe that, in this way, a quantum of mischief is committed, and a quantum of misery inflicted far exceeding belief. We wish we could assert that none of the regular faculty ever compromised the dignity of the profession, or forgot their own rank in society, by setting the example of manœuvering but a degree removed from the practice of empirics themselves!

Mr. Farr introduces a short, and we imagine, a pretty correct, account of the different means, regular and irregular, now in use for the relief or cure of cancer. He commences with the pressure process of Mr. Young, which he considers to be ingenious, and indicative of mechanical talent in its inventor; but laments that this gentleman should not have confined his plan to those cases where it was likely to prove useful, as he would then have sustained the credit and reputation he gained at its first introduction. We foresaw, indeed, that when Mr. Young had got so enamoured of his favourite measure as to make it paramount, or even exclusive in the treatment of open cancer as well as scirrhus, his plan would soon fall to the ground, as it has now done. We have reason to know, however, that the pressure, applied by himself and a few others who followed carefully his exact mode of application, was, in some instances, successful, and in several productive of relief. But the "cancer-institution" is now no more, and its founder, we believe, has left this metropolis.

Mr. Lloyd, of Falcon Square, appears, Mr. Farr observes, to follow, with some variations, the method proposed by Mr. Carmichael, using, in addition, setons near the diseased part, and occasional sedatives.

Mr. Wheeler, of this city, is much in the habit of treating cancerous cases, (and he has a good deal of practice in that line) by hemlock internally and externally. Mr. Chevalier, of South Audley Street, it seems, follows the same plan.

There is a Dr. M'Donald, of Orchard Street, who treats cancer constitutionally, and, from Mr. Farr's account, has been successful in four cases. But we have now entered the domains of the nostrum-monger; for Dr. M'Donald, who is like the famous Captain Wattle—"*all* for love, and a *little* for the bottle"—throws a veil of mystery over his method of treatment, and "like the miser with his hoard, hugs himself in the thoughts of his possessions," without communicating, for the good of suffering humanity, this important secret. But we absolve this medical miser of the sin of withholding his treasures from the community, perfectly convinced that he has no such treasures to distribute.

After what appeared in a former number of this Journal, it will not be necessary to say much of the knight of Nelson Square, Mr. Aldis, who is running his career of glory in the nostrum-monger line. This worthy member of the College of Surgeons, had the effrontery to publish a book on a *secret* remedy, and dedicate that wretched performance to the late President of the College of Physicians! Nay, he had the audacity to address the President in terms of familiarity, as "My dear Sir," and use other expressions that might lead the public to conclude he was actually acquainted with Dr. Latham, or that Dr. Latham had given him permission to dedicate this puff to him! Sir Astley Cooper's name is also profaned by the draw-cansir knight.

We have said before, that some degree of sanction or precedent has been given to the nostrum-monger, by certain imprudent members of the regular profession pretending to *peculiar*, and, in some instances, *concealed* modes of relieving or curing diseases. We are decidedly adverse to all such pretensions, as being not only derogatory from the dignity of a liberal science, but calculated to draw ridicule on the profession itself. It is painful to us to notice these aberrations from prudent and dignified conduct; but we deem it a duty to throw out these hints, and hope they will be taken in the proper quarter.

We shall here also make a few reflections on the subject of suppressing quackery, and restraining irregular practitioners. The College of Physicians have been blamed for permitting Charlatanism to flourish under the very eye of the College. But we really know not how that Body can undertake the dirty and Herculean task of cleansing this Augean stable, while the Government lends its sanction towards the accumulation of the filth. We observe, with grief also, that almost every Charlatan in London belongs, or asserts that he belongs to, the College of Surgeons! Not one of them dares to make the same use of the College of Physicians. Here then is a most convincing proof of the great utility which has resulted from the rigorous laws of the latter body, since not a single member of that College has disgraced himself by empirical conduct. So far, indeed, from wishing to see the College laws relaxed, we sincerely hope they will be made still more inflexible. We are quite satisfied that were the facilities of entering the portal of Warwick Lane as great as those attending the passing under Machaon and Podalirius in Lincoln's-Inn Fields, we should soon have every newspaper teeming with advertisements of *secret remedies* invented and sold by "members of the Royal College of Physicians of London." Let the College of Surgeons ponder on these things; and let the College of Physicians watch, with a jealous eye, the admission of members who have shewn any disposition towards secret remedies, or concealed processes in the healing art.

There is yet another subject to be noticed, and that is—the toleration by the College of several medical gentlemen (regularly educated, but not members of the College) within the metropolis, who officiate as physicians, and take the title of doctor. Reflection and observation induce us to think that it is both wise and liberal in the College to abstain from every thing in the remotest degree resembling persecution, or, what would be the same thing, *prosecution* of the said gentlemen. The present state of society, intellectual and moral, would render any such measure both odious and nugatory, even were the College inclined to exercise it, which we believe they are not. The College very properly stands on the *defensive*, and says—" thus far shall ye come, but no farther," unless ye fulfil the prescribed conditions. We shall take no step to prohibit your practice, but we will not consult with you, nor acknowledge you as members." It is abundantly evident that no man can hope for any thing like practice, *as a physician*, in London, with such a millstone round his neck. And if a man chuses to practise midwifery, or surgery, or pharmacy, or all three, and yet retain the title of M. D. conferred by another university, we do not see why the College of London should entangle itself in law-suits to compel these individuals to relinquish mere *titular* dignities which are not even the shadows of substantial benefit to themselves, or practical encroachments on the prerogatives of others. We repeat it, therefore, as our decided opinion, that the College is perfectly right, and perfectly politic, in abstaining from every other measure than the veto of consultation, in the cases under consideration. And knowing, as we do, the high respectability and superior attainments of some of the individuals thus circumstanced, we consider it both unjust and ungenerous, in any of their brethren, to hold up *that* as a stigma on their professional character which the only proper authority, the College itself, has not thought proper to visit with its censure.

The fucus helminthocorton, the subject of the present Essay, need not detain us long. It seems that, in a conversation between Mr. Farr and Mr. O'Meara, the latter recollected some observations of Napoleon, (a pretty medical authority truly!) expressive of his surprize that " Mousse de Corse," which was employed on the Continent merely as an anthelmintic, should not be employed also in the dispersion of tumours, as he had seen instances of its efficacy in the Island of Corsica. This hint was not to be lost, and accordingly our author procured the helminthocorton which, as a matter of course, " has more than answered the expectations he had at first formed." P. 13. Mr. Farr acknowledges that open cancer is not to be cured by the new remedy. It is only applicable to some forms of "*occult cancer.*" We shall occupy our readers' time no longer on this subject; for it is hardly worth while to "prognosticate a prophecy," as the Morning Chronicle used to say, respecting the fate of the helminthocorton.

